# Drought-induced soil microbial amino acid and polysaccharide change and their implications for C-N cycles in a climate change world

**DOI:** 10.1038/s41598-019-46984-1

**Published:** 2019-07-29

**Authors:** Madhavi L. Kakumanu, Li Ma, Mark A. Williams

**Affiliations:** 1 School of Plant and Environmental Sciences, 301 Latham Hall, Virginia Polytechnic and State University, Blacksburg, VA 24060 USA; 2 Present Address: Department of Entomology and Plant Pathology, North Carolina State University, Raleigh, NC 27695 USA; 3 Present Address: USDA Salinity Laboratory, Riverside, CA 95616 USA

**Keywords:** Carbon cycle, Microbial ecology

## Abstract

High microbial carbon (MBC) demand, a proxy for energy demand (cost), during soil microbial response to stressors such as drought are a major gap in understanding global biogeochemical cycling of carbon (C) and nitrogen (N). The dynamics of two dominant microbial pools (amino acids; AA and exopolymeric substances; EPS) in soils exposed to drying and C and N amendment to mimic both low and high nutrient soil habitats were examined. It was hypothesized that dynamics of EPS and AA (osmolytes) would be greater when soil drying was preceded by a pulse of bioavailable C and N. Drying reduced AA content, even as overall soil MBC increased (~35%). The increase in absolute amounts and mol% of certain AA (eg: Taurine, glutamine, tyrosine, phenylalanine) in the driest treatment (−10 MPa) were similar in both soils regardless of amendment suggesting a common mechanism underlying the energy intensive acclimation across soils. MBC and EPS, both increased ~1.5X and ~3X due to drying and especially drying associated with amendment. Overall major pools of C and N based microbial metabolites are dynamic to drying (drought), and thus have implications for earth’s biogeochemical fluxes of C and N, perhaps costing 4–7% of forest fixed photosynthetic C input during a single drying (drought) period.

## Introduction

Soil drying and drought are considered major disturbances that effect the physiological function of microbes that can resonate to global scale carbon (C) and nutrient biogeochemistry. Relative to a soil maintained moist, the enhanced abundance of soluble C, CO_2_, and nutrients following the re-wetting of a dry soil have been observed to persist for days and cost the equivalent of up to 5%, per event, of inputs coming from net primary production in a grassland^1^. Drying and drought are therefore considered a major driver of C, and by extension, energy flow in ecosystems that could shift the balance of terrestrial and atmospheric C and nitrogen (N) balances.

Data, indirectly, tend to support the idea that microbial production of physiologically expensive organic osmolytes help to explain the large pulses of CO_2_ when dry soils are wetted^1–5^. A number of recent studies have attempted to more directly link the pulses of microbial respiration, and related flushes of organic C and nutrients following re-wetting of dry soil to that of microbial production of organic dessication–protective osmolytes to cope with water potential stress, but these soil-based studies often conflict^5–10^ with expected organism responses^11–13^. Direct molecular evidence to refute or support the Osmolyte Accumulation Hypothesis (OAH) and the potential C and N effects during soil drought need further study to assesses their potential impacts on global nutrient dynamics

It has been difficult to explain the C and N dynamics of the dry-rewet flush. A high percentage of microbial C (energy) and large amounts of reduced bioavailable C would be needed to explain microbial adaptation to water dynamics using an osmolyte model paired with estimates of plant C flow^14,15^ and microbial biomass turnover^1^. There is thus a need to better understand the mechanisms that drive microbial adaptation and nutrient flux that occur directly following soil drying^4,7,16^. The adaptation of microbes to water potential decline, for example, by AA-osmolyte production, can also be used to build cellular proteins, comprising ~50% bacterial cell dry weight and a similarly large component of soluble organics in soil. The structural backbone of many osmolytes and osmolytes themselves in general are amino acids (AA). For example, neutral AA, proline (Pro), glutamine (Gln), glutamic acid (Glu), and taurine are oft described microbial osmolytes^17^. Polysaccharides (and proteins), similarly, exuded from the cell membrane are used to create exopolysaccharides and extracellular polymeric substances^11,18^ that support microbial adaptation to dessication. To exemplify this latter response, upon starvation or other stress, gram-negative *Myxoccoccus xanthus* will form a polysaccharide spore-like fruiting body of ~10^6^ cells^19^, and so like many other biofilm forming bacteria utilize polysaccharides to cope with stresses and then regain activity when environmental conditions allow. The nature of these adaptations at the small scale could have wide spread implications at the larger biogeochemical scale, especially in response to changing climate.

Biologically available C and nutrients are generally low in soil, but this generalization ignores high heterogeneity, especially at the milli- and micro-meter scale where plant roots and rhizosphere, for example, play a dominant role in support of heterotrophic activity and plant-microbial interaction^20–22^. The heterogeneity almost certainly exists as multiple gradients from relatively low to high bioavailable pools of C and nutrients but can be simplified conceptually into two habitats representative of low and high zones of heterotrophic activity. The production of osmolytes during low water potential has been shown to be highly regulated by the presence of organic molecules and nutrients^23^, therefore, the availability of C and N near the root-zone^5^ may help determine how soil microbes respond to variability of water availability and water deficit stress, and thus may help to explain the variable reports explaining microbial responses to soil drying.

It was hypothesized that greater availability of C and N would increase energy and nutrients to change the type, and increase the amount of putative intracellular organics AA; Gln, Glu, Pro, taurine and polysaccharide-type biofilm production to cope with soil drying, especially in a soil prone to drought. Studying the dynamics of these molecules can be used to describe, relatively speaking, energetic demands needed under either oligo- or copiotrophic soil conditions. To test this, an experiment was conducted with two very different soils, a lowland Marietta (silt loam), which is relatively moist and with fewer natural swings in water potential; and an upland Sumter soil (clay loam), with frequent and large swings in water potential^6^. Soils were exposed to different levels of matric water potential deficit (−0.03 MPa, −1.5 MPa, and −10 MPa) following simulated rhizosphere inputs of C-N (carbon and nitrogen amendment), C only (carbon only), or no amendment (water-only). An assessment of the potential C used to cope with osmotic stress in forest ecosystems is also presented to help account for the potential costs of coping with drying and drought.

## Results

### Microbial amino acids (mass and mole percentage)

**Figure 1:**
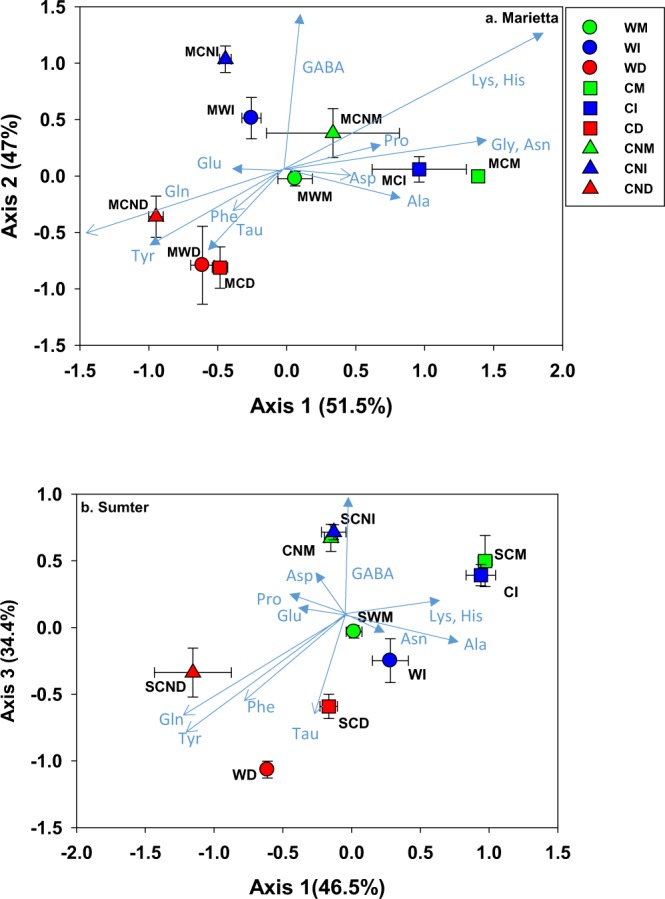
Nonmetric multidimensional scaling plot showing the mol% distribution of microbial amino acids sampled across a gradient of matric potential and nutrient amendment (**a**) Marietta and (**b**) Sumter. Shapes designating different nutrient amendment are shown, and drying treatments are designated by different color: Moist (Green), Intermediately dry (Blue) and Dry (Red). Simultaneously, first letters in the labels indicate nutrient amendment: No amendment (W), Carbon only (C), and C and N (CN) followed by drying treatment indicated by Moist (M), Intermediately dry (I) and Dry (D). The shapes and lines represent the average and standard error (n = 3) for drying and amendment treatments. Vectors that are correlated with each axes (r > 0.65) are shown. The direction and length of each vector represents the treatment association and strength of the correlation across axes. Percentages denote the proportion of variability associated with each axis (McCune and Mefford, 2011). Individual Multi-response permutation tests detected strong significant differences between soils, and so each soil is depicted separately. The main factors of amendment, and degree of water deficit (p < 0.01) are shown for each soil. An interaction was detected between amendment and degree of water deficit (p < 0.01).

The mole percentage (mol%) of AA in Sumter and Marietta soil were, as expected, very different (MRPP; *p* < 0.000001) and are thus analyzed and presented separately to highlight the differences associated with amendment and drying. Both amendment and drying significantly affected the distribution of mol% AA (MRPP; *p* < 0.00001). In response to the most extreme (−10 MPa) drying, the mol% AA in both soils indicated similar patterns of variation (Fig. 1). The shift of AA in similar ways in two different soils suggests comparable mechanisms of microbial acclimation to drying. Yet, despite this similarity, the overall mass of AA was ~ 2X greater in Marietta than Sumter (MRPP: *p* < 0.00001), thus suggesting the size of the response may depend on the size of the microbial biomass. Each soil, moreover, was affected similarly to the interaction between amendment and drying (MRPP; *p* < 0.001). Though each soil initially had a rather different mol% and dominance of AA, their shifts to drying and amendment were consistent (Fig. 1). The similar dynamics in AA mol% due to drying in both soils suggest similar microbial physiological acclimation.

Axis 1 of the NMS plots were positively correlated (r > 0.70) to Gln, taurine, tyrosine (Tyr), and phenylalanine (Phe), and negatively with lysine (Lys). Glycine (Gly) and histidine (His) responded similarly to that as Lys, but were correlated below the threshold of r > 0.70. Similarly, low Gamma-amino butyric acid (GABA) concentration in dry relative to moist soils may suggest possible stress response due to drying. Likewise, both soils showed AA shifts in the moist and moderately dry soil that was closely associated with both drying regime and amendment type. Along Axis 2, GABA tended to increase with C and N amendment in relatively moist soils, but the effect was larger for Marietta compared to Sumter. GABA also was ~2–3X lower in the dry compared to moist soils (*p* < 0.05). Lys and His mol% were positively correlated with C only amendment. Overall both soils showing similar shifts in AA in response to the most extreme drying (Fig. 1).

**Figure 2:**
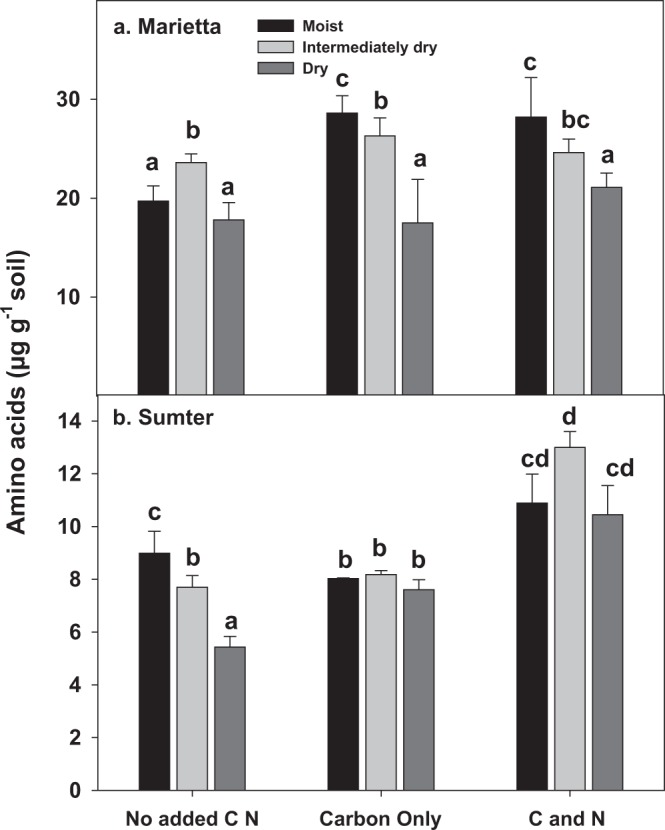
The amounts of microbial AA (µg g^−1^ soil) in two soils, Marietta and Sumter, at different levels of matric potential deficit tended to decline; the C or C and N amendment increased AA pools, but to a lessor degree than the effect of soil drying. Bars represent the mean of 3 replicates and line the standard error, respectively. Treatments not having the same letter are significantly different (α = 0.05) within each soil type. The total AA abundances showed strong significant differences between soils (p < 0.0001), and so each soil is depicted separately. Degree of water deficit, amendment, and amendment by water deficit interaction were shown to have significant effects on AA abundances (p < 0.05).

The total AA pool sizes of C only and C-N amendments increased by ~ 25% in Marietta (*p* < 0.01) in the moist soil (Fig. 2a), but the pool sizes decreased as a result of drying (*p* < 0.01). The exceptions were in the unamended Marietta and C-N amended Sumter, where AA were greatest in the moderately dry relative to dry and moist treatments. C-N amendment but not C only, also increased the level of AA by 50% in Sumter soil. The overall decline in AA associated with microbial pools in the dry soils have been observed previously and suggest a mechanism of microbial adaptation to soil drying^6^.

### Absolute amounts and changes in specific microbial amino acids

Glutamate pool sizes were the largest, accounting for ~38–58% of all AA (Supplementary Fig. S1 and Supplementary Table 1). Alanine (Ala) and Leucine (Leu) accounted for ~8% each across soils and most other AA ranged from 2 to 5%. There were however, large (6 to 10 X) differences in Gln, GABA, and Arginine (Arg) which accounted for 9%, 7% and 5% respectively of total AA in the Sumter, and less than 1% in Marietta soil (Supplementary Table 1). In contrast, Marietta tended to have higher concentrations of other AA, with 5 X more Pro and His, and ~1.5–2.5 X more of Aspartic acid (Asp), Threonine (Thr), Ala, and Valine (Val) relative to Sumter, respectively, each accounting for approximately 5, 4, 10, and 9% of AA pools. Microbial AA are also shown per unit of microbial biomass carbon (MBC) (Supplementary Table 2), however, because changes in MBC during drying are thought to derive from a large and varied pool of osmolytes, this paper focuses on changes in AA per gram of soil.

**Figure 3:**
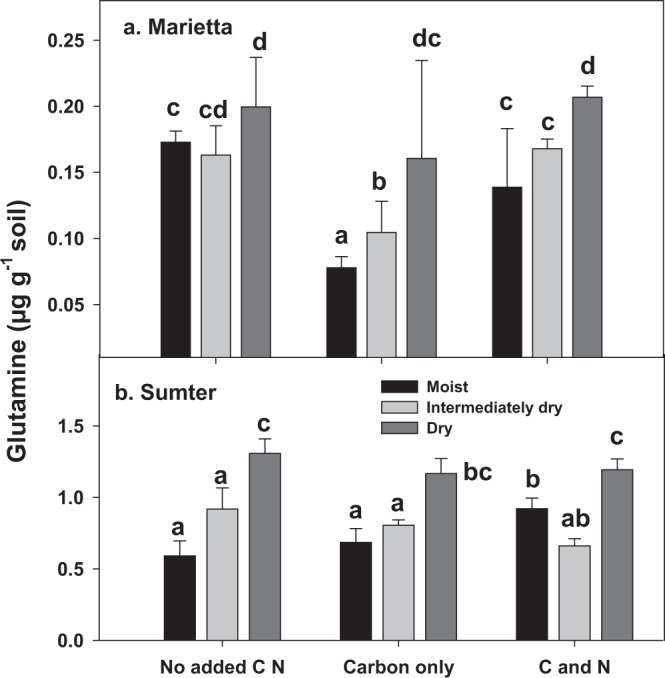
The amount of microbial extractable Gln (µg g^−1^ soil) in Marietta and Sumter soils changed at different matric potential deficits and due to amendment of C or C and N. Gln was positively affected by both drying and amendment in both soils. Strong significant differences between soils were observed (p < 0.0001), and so each soil is depicted separately. Bars represent the mean of 3 replicates and the lines represent the standard error. Treatments not having the same letter are significantly different (α = 0.05) within a soil. The glutamine values were significantly affected by soils, degree of water deficit, and amendment type (p < 0.05).

**Figure 4:**
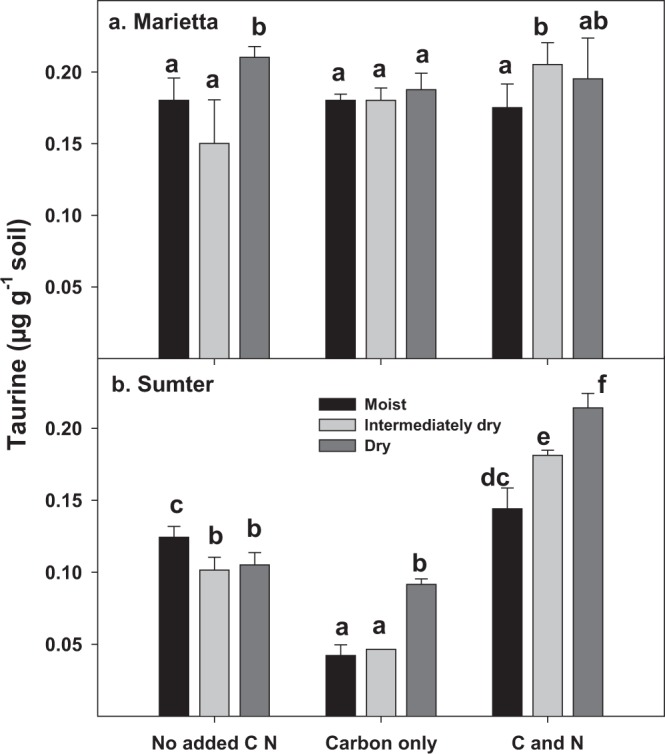
The amount of microbial extractable Tau (µg g^−1^ soil) increased in Marietta and Sumter soils at different matric potential deficits and due to amendment of C or C and N. Tau was positively affected by both drying and amendment in both soils. Strong significant differences between soils were observed (p < 0.0001), and so each soil is depicted separately. Bars represent the mean of 3 replicates and the lines represent the standard error. Treatments not having the same letter are significantly different (α = 0.05) within a soil. The Tau values were significantly affected by soils, degree of water deficit, and amendment type (p < 0.05).

Glutamine, a putative osmolyte, was the only proteinaceous AA that consistently increased in absolute abundance due to the most extreme drying (−10 MPa) across both soils (Fig. 3). Taurine increased due to soil drying, and the response to drying was greater following C only or C-N amendment (Fig. 4). In the Sumter, but not the Marietta, Pro (Supplementary Fig S2) also increased per gram of soil when dry soils were amended with C-N, and thus showing some support of the main hypothesis that osmolyte production during drying is limited by C and N. However, it should be noted that the results are presented as ng osmolyte g^−1^ of MBC (Supplementary Table 2a,b), no increases in putative AA osmolytes were observed as a result of drying or drying X amendment. There were, however, significant reductions in most AA due to drying. The results thus support the idea that C and N had an effect on AA or AA-osmolyte pools but the size of these pools was relatively small as proportion of all AA.

### Shifts in microbial carbon

**Figure 5:**
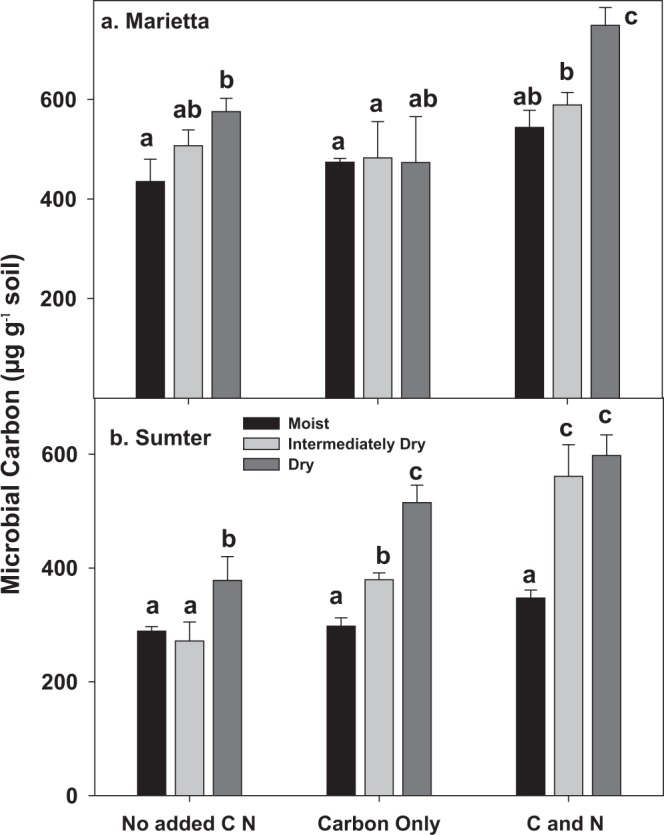
The amount of microbial extractable C (µg g^−1^ soil) in two soils, showing the strong positive effect of drying and to a lesser extent amendment in Marietta and Sumter soils. Bars represent the mean of 3 replicates and the error terms represented by the standard error. Strong significant differences between soils were observed (p < 0.0001), and so each soil is depicted separately. Treatments not having the same letter are significantly different (α = 0.05) with each soil. Microbial C abundances were significantly affected by soils, degree of water deficit, amendment type, and amendment x water deficit interaction (p < 0.05).

Microbial biomass carbon (MBC) increased only moderately to the nutrient amendment (~10–30%), however when drying was coupled with amendment, MBC increased the most (30 to 45%), (*p* < 0.001) indicating an interaction between drying and amendment (Fig. 5). Microbial biomass also increased (*p* < 0.001) in both soils within each amendment as a result of drying. There is thus an accumulation of osmolytes or other cell constituents due to drying, which was enhanced especially if drying was preceded by C only in the Marietta and by either C only or C-N amendments in Sumter soil (Fig. 5), respectively. Overall, the results indicate that MBC, and possibly other organic osmolytes or extracellular biofilm increased due to the most extreme drying, especially when the availability of C and/or N was relatively high. This results support the hypothesis that C and N supply can limit microbial response to drying.

### Shifts in polysaccharides due to amendment and drying

**Figure 6:**
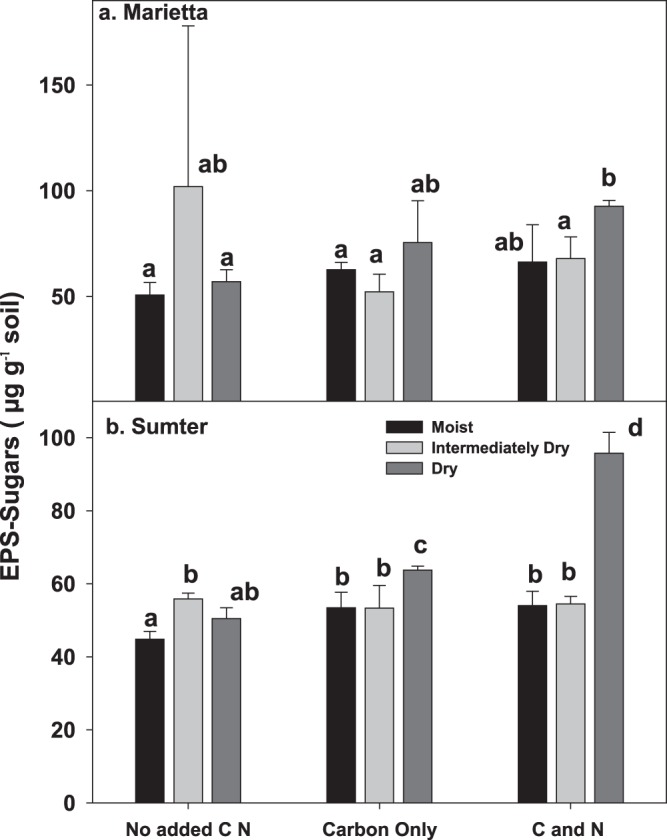
The amount of (exopolymeric) EPS-sugar (µg g^−1^ soil) in two soils, Marietta and Sumter, showing strong effect of levels of matric deficit interacting with amendment. Both soils showed variation in EPS in response to drying, but the most positive effects were observed in the drought-prone Sumter soil, Bars represent the mean of 3 replicates and the error term represented by the standard error. Strong significant differences between soils were observed (p < 0.0001), and so each soil is depicted separately. Treatments not having the same letter are significantly different (α = 0.05) within each soil. The amount of EPS–sugars were significantly affected by degree of water deficit, amendment type, and amendment x water deficit interaction (p < 0.05).

Concentrations of microbial exopolysaccharide (**EPS;** Supplementary Fig. 3) only increased marginally, if at all, with amendment. When amendment was coupled with drying, however, there were increases of 30 to 70% relative to moist or unamended soil (*p* < 0.001). The largest changes due to the most extreme drying, especially in the Sumter soil, occurred following C-N amendment (Fig. 6). These results suggest that microbial EPS may be a mechanism of microbial adaptation to soil drying, especially when copiotrophic conditions persist.

## Discussion

As the primary conduit of net C loss from soil, the response of microbial communities to drought and drought following nutrient amendment in soil can be viewed functionally as specific changes in the absolute abundance or relative mol% changes of microbial cellular contents (molecules) such as osmolytes. It should be noted that we have not measured the full suite of osmolytes, but rather a pool related to the largest group of potential osmolyte molecules, amino acids. Betaine, ectoine, and glycine-betaine are examples of other osmolytes observed in soils but that were not measured here^10^. Regarding both the absolute abundance or relative mol% changes, patterns of microbial AA change in two soils were grouped as: (1) those specifically highlighting the most extreme drying as an overriding effect regardless of amendment, (2) under moist or intermediate drying conditions, C only and C-N amendment were each associated with specific mol% AA changes (Fig. 1); and (3) some AA and extracellular polymeric pool sizes increased in response to the most extreme drying, as expected, but primarily when C-N were amended. Overall these results suggest a consistent microbial physiological AA response to drying, regardless of soil type.

There were effects related to drought-prone relative to the mesic soil, however, and the former did have a greater overall putative AA-osmolyte response than the mesic soil, despite having a smaller microbial biomass. The most notable microbial AA changes due to drying were absolute (µg g^−1^ soil) decreases in overall AA pools, and mol% increases in the known osmolytes like Gln, taurine and lessor known osmolytes such as Tyr, and Phe. Similarly, there were mol % decreases in Lys, His, and GABA. The biogeochemical ramifications of this putative general stress response of AA and EPS suggest that soil microbes might share some common physiological adaptations to drying and drought across multiple soil and ecosystem types.

An important point that is often not discussed in soil experiments testing for the role of osmolytes as mechanisms of microbial adaptation to soil drying is whether the changes in osmolytes should be based on a per gram of soil or per microbial biomass basis^6,9,22,24,25^. Indeed, our results often show increases in osmolytes g^−1^ soil, but not g^−1^ microbial biomass. It is thought that the former rather than the latter are most reported as a consequence of the tendency for large increases in microbial biomass when soils are dried. The increase is not thought to be due to growth, because growth would be expected to slow down during drying, and is hypothesized to be the result of increasing pools of osmolytes within the cell^2–7^. In this regard, a bias of the microbial biomass method is that it is thought to extract more intracellular than cell wall and wall associated material^21,22,24^, and therefore may represent less than half of the actual microbial biomass. The fact that microbial biomass measurements vary widely, are often 100X the size of a specific osmolyte, and tend to have variability that are greater than the size of an osmolyte pool would also overwhelm changes in osmolytes and likely be a factor in reporting. Hence, a g^−1^ soil basis is a standard that can be compared across studies, especially when microbial biomass is not reported, or when different microbial methods are used. Solutions to this conundrum will need to be determined in order to assess the role of osmolytes among soil microbes.

Though not indicative of absolute change, the highly significant mol% shifts in microbial AA should not be ruled out as indicators of osmotic adaptation. Shifts in the chemical mixtures of molecules such as that shown for Gln, taurine, and Tyr, can have powerful non-linear effects that can help to explain microbial adaptions using OAH. Perhaps more important regarding C and N dynamics, and associated energetics is the need to more completely understand the mechanism of soil microbial response to water deficit. It is important to recall that as microbial AA pools tended to decline with similar patterns of change in both soils, the microbial biomass C pool, perhaps related to intracellular non-AA osmolytes, tended to increase by ~30% due to drying^26^. This consistent change across soils further suggests an important microbial physiological change, and the potential importance of cellular re-allocation and osmolyte accumulation as a means to acclimate to soil drying.

Not all AA are thought to act as strong osmolytes, but notably, an increase (per gram of soil) in the common osmolytes like Gln, taurine, and to some extent Pro as a result of drying were consistent with OAH^27,28^. Glutamine, in particular, was relatively abundant, and increased due to drying in the Sumter soil. Overall, these AA make up <1 to 5% of the total AA measured in microbial cells, and thus may not be considered significant enough to offset the osmotic challenge of soil drying, however, the majority of microbes in soil are considered inactive or dormant and so these AA increases could, in fact, be ecologically significant^8,9^.

The consistency of the amino acids that decreased in putative microbial cytoplasmic pools are also important to note because they suggest similar biological acclimation across soils and may indicate the potential for polymerization to form proteins or integration into cell wall constituents and/or the breakdown and transport of cytoplasmic proteins for other cellular uses. There is also the potential for dormancy or sporulation, and the need for the synthesis of picolinic acid (PA), that would then result in a decline in Lys, a major precursor to PA^29–31^. There are thus multiple means to explain the consistent dynamics of AA in response to drying. Based on the ~25% decline in total AA, this is considerable turnover of the major building blocks of microbes. The internal dynamics of cellular AA (fungal and bacterial) are likely only part of the story of microbial biomass response to drying, and declines in AA may be part of acclimation. When re-wetting (e.g. rainfall) a dry soil, the AA and reduced C (energy) needed to respond to this instantaneous change in water potential in a drying soil may help prepare the cell (adaptation), ultimately, for the expected return of rainfall and wetting.

The role that aromatic proteinaceous AAs (Tyr, Phe) play in microbial adaptation to relatively extreme soil drying deserves further inquiry. Overall, mol% increase in these AA fit with the role that neutral AA are thought to play as stabilizers of cytoplasmic proteins^11,13,17^. Thus, though they may not be osmolytes, per se, they could provide a supportive role as protein protectants. Tyrosine is not often reported as an osmolyte of microbial cells. It is, however, described as an important component of proteins that are involved in intracellular communication^32^. Phosphorylation of proteins rich in Tyr, for example, play key regulatory roles in bacterial physiology, linked to exopolysaccharide production, stress response and substrate phosphorylation^18^. Free Tyr found in the cytoplasm under dry and relatively high C-N conditions may represent shifting cellular activities under stress, and that require its production to form Tyr rich proteins used for microbial cellular adaptation. Taurine biosynthesis, furthermore, has been shown to be associated with cell wall phospholipid and slime production^33^. These AA may thus have a role in water stress regulated polysaccharride production.

Recent studies have measured the dynamics of numerous microbial metabolites such as AA, sugars, and alcohols in response to drying under controlled laboratory conditions, but tended to observe small shifts in putative osmolyte pools^1,6,8–10^. In contrast, mesocosms growing plants showed a 10 X increase in several known osmolytes (e.g. ectoine, betaine) following a 21 week drying period^5^. This latter study raised salient points about the difficulty of studying microbial osmolyte dynamics in soil. In that study, for example, it was not clear whether molecular changes in extractable osmolyte– type compounds were derived from plant or microbial sources. Differentiating these sources is important for understanding the soil microbial role in drought related biogeochemistry. At the same time, the study suggested that N related molecules, including proteinaceous AA are likely important in the soil microbial response to soil drying.

Microbial biomass carbon increases with soil drying have been documented previously, and may be one of the more consistent responses to drying^4,8,22^, and notably similar in type, to the mol% AA change. The results have been considered as support for the hypothesis of microbial osmolyte and/or EPS production in response to drought. Uptake of water soluble molecules that may become more concentrated around microorganisms as the volume of water declines during soil drying could also support microbial uptake^6,8^. In this case, it would be expected that there should be an increase in the intracellular pool of metabolites and potentially microbial growth. If this is true, then the increase in microbial biomass seen with drying may not be an adaption to drying and osmolyte production, but rather a response to greater available organics. Given the costs associated with acclimating to water potential decline^1^, the newly available C and energy provided during soil drying may be put to use to support microbial acclimation. These could include glycerol and mannitol (alcohols) and betaines which have been shown to increase with soil drying^5,6,10^. Indeed, drying may be a boon to mineral or other surface bound microbes because molecules would concentrate and flow to them. This could allow for the decline of some AA precursors and their transformation into some of these other types of osmolytes. The precipitous decline in Gly may have been the result of reallocation of precursors to form trimethylglycine using a rather unique pathway, a rather well known osmolyte, in lieu of Gly production^34–36^. Changes in microbial acclimation via C, N and energy use to drying deserve further scrutiny for understanding microbial acclimation to drought and how it may affect and respond to future climate change.

Peptide, protein and peptidoglycan formation have been shown to be important components of microbial osmotic hyper and hypo-osmotic responses^37,38^ and may thus also impact the size of soluble pools through cellular re-allocation from cytoplasm to cell wall. When in D-form and not the more typical protein based L-form, the AA Ala, Lys, and Glu (in D-forms) are common components of peptidoglycan and could thus be in relatively high demand for cell wall formation^39,40^ during soil drying. Though we could not differentiate the D and L-forms of AA, there were strong weightings on the NMS ordination axes indicating that microbial soluble Lys and Ala were negatively associated with drying. It is not possible to know if the pool size changes are related to declining free AA pools and subsequent integration into peptidoglycan, but this scenario is consistent with how microbial cytoplasm might change if high demand for cell wall production co-occurred with soil drying. More insights into the dynamic nature of the AA pool and cell wall changes would help to understand these microbial expenses under drought conditions.

Exopolysaccharides and exopolymeric substances (polysaccharides, proteins) provide a network surrounding bacterial populations and individual cells, and help to hold water, provide physical support to the cell wall, and help to contain the cytoplasm of bacterial cells^41–43^. EPS may thus help to protect cell integrity or reduce the impact of water deficit on soil microbes, *in situ*. Both soils showed evidence for EPS production when soil was dried and when C-N availability was relatively high but the Sumter soil responded more strongly to drying than the Marietta. This increase is consistent with the broad pattern of drying induced increases in microbial biomass. There is thus the potential for a large amount of production and reallocation of cellular organics in response to drying when C and nutrient resources are available, such as habitat adjacent to a root.

In contrast to expulsion and possible microbial osmolyte resource losses from a cell following re-wetting induced dilution stress, EPS may be energetically more favorable to osmolyte accumulation because it represents a resource attached and allocated to the cell wall perimeter. Hence, when soils are re-wetted, and cellular osmolytes released into the surrounding soil solution, they are potentially lost energy and nutrients for a microbe. EPS, in contrast, would be expected to maintain greater attachment and thus accessibility to a cell or fungal organism throughout all types of environmental change. Hence, protein production provides a mechanism to explain the observed reduction of the microbial intracellular AA pool, which often accompany the extracellular production of EPS. Though we did not explicitly measure protein as part of the EPS fraction, declines in AA could contribute to EPS production.

Large populations of bacteria can produce a network that may not be simple to differentiate into contributions from single cells. In this latter case, the gains of living in a population structure may stem from more efficient use of resources in the production of EPS, and allow members of the clonal population to adapt to water potential change and maintain cellular activity in an otherwise osmotically challenging environment^12,13^. The results support the hypothesis that EPS production can be a mechanism of soil microbial response to soil drying and possibly a greater part of acclimation in drought-prone habitats when C and N are not limiting, such as that near a root rhizosphere.

## Conclusion

This study showed results supporting the idea that microbial accumulation and reductions of specific AA could be a part of the mechanism used by soil microbes to respond to drying and EPS to drying that occurs following C-N amendment. However, because overall microbial biomass increased simultaneously as these putative osmolytes, this conclusion on a per gram of microbial biomass basis remains to be further verified. It is notable that the two soils did share similar mol% AA change in soil drying (e.g. Gln, Phe, Tyr, taurine), indicating that relative concentrations of AA may be important, or indicative of acclimation through AA reallocation across soils. EPS production provided the strongest indication of a microbial response to drying when associated with C-N amendment. In some cases, limitation and availability of nutrients and C in oligotrophic soil compared to that of copiotrophic habitats near detritus or plant root-zones may induce and ultimately determine the mechanism of microbial response to soil drying^5,8,44^. Carbon and C-N availability alter the microbial response to desiccation through changing EPS concentrations during drying and help to explain previous variation in microbial responses to drought across studies. In this case, the reliance of C and N amendment to the soil for the production of EPS is a novel finding, regardless of changes in microbial biomass. Overall, the even larger increases in total microbial C support the notion that microbial acclimation to drought can result in large shifts in microbial AA and EPS pools.

The collective dynamics in AA, EPS, and total microbial biomass, assuming these microbial pools are independent of one another, increase or decline in mass by 55–110% due to drying, with the higher end estimates exacerbated when preceded by simulated rhizosphere C-N flow. This indicates the potential for high turnover and a useful proxy for estimating energy use (in C units) as part of microbial acclimation to drought-type conditions. A reasonable estimate of net primary productivity (NPP) of the 50 to 100 year old forests in this study are 400 and 200 g C m^−2^ of aboveground leaf and belowground root input, respectively, each year. Though we only sampled the top 10 cm of soil, up to 20% of total root biomass can be found in this layer. With the measurement of 30 g of MBC per m^2^ in these surface soils, and 440 g C m^−2^ of new yearly plant input; would indicate that MBC change (16–30 g MBC/440 g plant C) is equivalent to ~4–7% of NPP-C. This is a slightly higher, but confirmatory estimate, than that for a grassland ecosystem^1^ in response to a single drying event. This estimate does not include N costs or the microbial response and turnover that results from dilution stress caused by re-wetting from rainfall, and thus may be a conservative estimate of costs to soil microbes. It should be noted, however, that if drying does increase microbial access to soluble soil organics through concentration of soil solution around microbial colonized soil particles, this large gain would help to greatly offset costs and also serve to explain the increase in MBC following soil drying. Internal recycling of organics can also serve to support microbial efficiency when dry soils are ultimately re-wet. Despite decades of study, further descriptions of microbial response to drying and subsequent re-wetting are needed to better describe the costs of global climate change (e.g. drought) to soil microbes and their impact on the broader ecosystem and global C-N balance.

## Materials and Methods

### Site description and soil collection

The experiment was conducted on two soils, Marietta and Sumter, located near Mississippi State University, Mississippi, USA (33°28′N and 88°47′W) in Fall 2009. The site description and soil properties were described in Kakumanu *et al*.^6^. Briefly, the Marietta soil is a fine-loamy, siliceous, active, thermic Fluvaquentic Eutrudepts derived from deep alluvial deposits near streams in the blackland prairie region of Mississippi. Marietta soils are located in the drainage areas of the mixed uplands of the Southern Coastal Plain and subjected to frequent flooding and with moist water status^45^. The Sumter soil is a carbonatic, thermic Rendollic Eutrudept, silty clay formed in marly clays and chalk of the black land prairies. It is a moderately deep, well drained, upland with medium granular structure and rapid runoff. The water table is deep and the permeability of the soil is slow^46^.

Top soil from ~10 cm depth was collected at 0, 50, and 100-m from three locations along a 100-m transect at each of the 2–5 Ha forested soil types. Soils were sieved through 4 mm mesh, thoroughly cleaned of obvious plant litter and rocks, and stored at 4 °C.

### Experimental setup

A laboratory experiment was conducted to determine the metabolite and microbial pools in response to drying following the amendment of C and N. Moist soil was weighed (25 g dry weight) into sterile 150 ml specimen cups, and allowed to incubate moist (−0.03 MPa) over a 3 d period. Either C only (62.5 mg of glucose) or C-N (62.5 mg of glucose and 8.99 mg of ammonium nitrate) solutions were amended to soil (C:N ratio = 8) to support the activity and growth of microbial biomass. No external nutrients were added to the other set of samples and maintained at −0.03 MPa similar to nutrient amended soils.

Specimen cups were immediately placed into 1 L canning jars and sealed with lids fitted with septa. Rates of CO_2_ production were monitored and used to estimate when to open lids on jars for aeration to maintain O_2_ concentration >18% (22 °C). Microbial growth was estimated to last up to ~96 h, as CO_2_ production approached an asymptote indicative of the transition from logarithmic growth to stationary phase. An estimation based on the difference between the amended and the unamended soils showed that 58 and 51% of the added C had been respired from the Marietta and Sumter soils over those 4 d, respectively. Specimen cups were removed from mason jars and soil allowed to slowly dry at a constant rate for 6–8 hours per day to simulate the natural change in water potential until reaching −1.5 MPa and −10 MPa. The driest sample took 3d to reach the target water potential but only one day for the sample to reach −1.5 MPa. Samples assigned to moist treatments were maintained at −0.03 MPa. Water uptake and transpiration near a root often ceases as soil approaches −1.5 MPa, but water loss near the soil surface on hot sunny days quickly exacerbate losses due to evaporation^47–49^. Rates of CO_2_ production in the moist soils amended with C and N approached the rates of the continuously moist soils that remained unamended, suggesting a steady state of respiration had been reached.

### Microbial biomass carbon and amino acid analysis

One day after reaching the target soil water potential, soil samples were extracted for MBC and AA according to Kakumanu *et al*., (2013), based on chloroform lyses of microbial cells and the consequent release and dissolution of intracellular cytosol into K_2_SO_4_ solution. Briefly, microbial molecules from 10 g (dry weight) of soil from each treatment were extracted using a mixture of chloroform and 0.01 M K_2_SO_4_ (1:4; v/v). After series of steps including stirring, centrifugation, and filtration, the supernatant solution was lyophilized and stored at −80 °C until further analysis. The dried residue was reconstituted in 1 mL of water and 10 to 50 µl aliquots were drawn for analysis of sugars, ninhydrin N, and AA.

Soluble sugars in the extracts were determined by phenol-sulfuric acid analysis (PSA)^50^ to determine MBC. Briefly, 15 µl of the re-dissolved soil extracted pellet was added with 50 µl of 80% phenol solution followed by addition of 5 ml of concentrated H_2_SO_4_ (~18 M). The mixture was incubated for 45 min at room temperature and the absorbance was measured at 480 nm. Glucose was used as a calibration standard for the analysis and MBC was calculated^51^.

Microbial AA were analyzed as previously reported^52,53^. The lysate was amended with 20 mM NaN_3_ and then spiked with 8 μL 2.5 mM internal standard, α-Aminobutyric acid (AABA) to account of AA recovery. Distilled water was added to bring the final volume to 10 ml of 10 mM K_2_SO_4_ and 10 mM NaN_3_. The mixture then was shaken at 100 rpm on a reciprocal shaker at room temperature for 15 min, followed by centrifugation at room temperature for 15 min at 4000 g. Following centrifugation, the supernatant was collected and filtered through 0.22 μm polyvinylidene fluoride (PVDF) membrane and an aliquot of exactly 500 μL of the filtrate was taken for centrifugal vacuum drying. The dry aliquot was derivatized using the AccQ Fluor reagent kit (Fluorescent 6-Aminoquinoly-N-Hydroxysuccinimidyl Carbomate derivatizing reagent; Waters Co. Cat# Wat052880) following the standard protocol from Bosch *et al*.^54^ and Hou *et al*.^55^. Chromatographic separations on the HPLC were carried out in a Waters X-Terra C18, 3.5 um, 2.1 × 150 mm. The column was set to 37 °C and the flow rate was 1 ml/min. The mobile phase A consisted of 170 mM Sodium Acetate at pH 5.05. Detection was carried out by fluorescence (λ excitation 250 nm and λ emission 395 nm) and quantified against a standard for each AA.

### Polysaccharide extraction and analysis

Polysaccharides from 10 g of soil was first extracted with 10 ml of 0.01 M K_2_SO_4_ by vortexing for 10 min and then centrifuged at 12000 X g (4 °C) for 10 min (Modified Chang *et al*., 2007)^42^, and included a heating step^43^. The supernatant was saved, and the soil was again treated with 10 ml of 0.01 M K_2_SO_4_ solution to boiling (~100 °C). Samples were vortexed for 1 min and vortexed again every four minutes in between boiling. After 20 minutes, the samples were cooled to room temperature and centrifuged as described above. The supernatant was removed and stored along with the previous supernatants. To the collected supernatant, twice the volume of ethanol was added and allowed to precipitate overnight at 4 °C. Samples were centrifuged, pelleted (Supplementary Fig. 3), the eluate removed, and the pellet washed. The pellet was re-suspended in de-ionized water and a portion taken to determine polysaccharide content using the phenol sulfuric acid method as described above for microbial biomass C. Though polysaccharides from non-microbial sources are likely to be assayed using this method, it is the change in polysaccharides that are of greatest interest, and assumed to come predominantly through microbial adaptation to water deficit.

### Statistical analysis

Initially, statistical analysis (3-way ANOVA) was conducted to analyze the effects of soil, nutrient amendment, and drying and their interaction on the response variables including AA, polysaccharides, and MBC. In all cases, as expected, soil had a large relative effect and therefore data were re-run separately using two-way ANOVA to analyze the factors of nutrient amendment and drying. All tests were considered significant at *p* < 0.05 (SAS 9.2, Sysstat software, 2008). Multiple comparisons were conducted using Fisher’s LSD. Tests of differences in normality (Shaperio –Wilk) and variance were non-significant. Multivariate analysis of the AA data were similarly shown to have a large soil effect (p < 0.001) and soils were thus analyzed separately, using PC-ORD version 6 software (MJM Software, Gleneden Beach, OR)^56^. The Multi-response Permutation Procedure (MRPP) was used to test for significant differences between multivariate data. Nonmetric multidimensional scaling (NMS) was used to provide graphical ordination of the mol% of AA data.

## Supplementary information

Supplementary Information

## Data Availability

The authors agree to the terms of making data available to the scientific community as defined by the Journal Scientific Reports.
