# Supplementary material for: Drought-induced soil microbial amino acid and polysaccharide change and their implications for C-N cycles in a climate change world
Source: Sci Rep. 2019 Jul 29;9:10968. doi: 10.1038/s41598-019-46984-1 (PMC6662807; doi:10.1038/s41598-019-46984-1)
Supplement: Supplementary file 1 — Supplementary Information [file 41598_2019_46984_MOESM1_ESM.docx]

**Drought-induced soil microbial amino acid and polysaccharide change and implications for C-N cycles in climate change world**

Madhavi L Kakumanu^1,2^, Li Ma ^1,3^, Mark A Williams ^1^*

^1^ *School of Plant and Environmental Sciences, 301 Latham Hall, Virginia Polytechnic and State University, Blacksburg, VA 24060*, USA*.*

*^2^Present Address: Department of Entomology and Plant Pathology, North Carolina State University, Raleigh, NC, 27695, USA.*

*^3^Present Address: USDA Salinity Laboratory, Riverside, CA, 95616, USA*

*Corresponding author: Tel 540-231-2547, Fax 540-231-3083, Email markwill@vt.edu

**Supplementary Figure 1**

Supplementary Fig: 1.The amount of microbial extractable glutamate (µg g^-1^ soil) in Marietta and Sumter soils at different matric potential deficits and due to amendment of C or C and N. Bars represent the mean of 3 replicates and the error term represented by the standard error. Strong significant differences between soils were observed (p<0.0001), and so each soil is depicted separately. Treatments not having the same letter are significantly different (α=0.05) with each soil. The glutamine values were significantly affected by degree of water deficit, amendment type, and amendment x water deficit interaction (p<0.01).

**Supplementary Figure 2**

Supplementary Fig: 2.The amount of microbial extractable proline (µg g^-1^ soil) in Marietta and Sumter soils at different matric potential deficits and due to amendment of C or C and N. Bars represent the mean of 3 replicates and the error term represented by the standard error. Strong significant differences between soils were observed (p<0.0001), and so each soil is depicted separately. Treatments not having the same letter are significantly different (α=0.05) with each soil. The glutamine values were significantly affected by degree of water deficit, amendment type, and amendment x water deficit interaction (p<0.001).


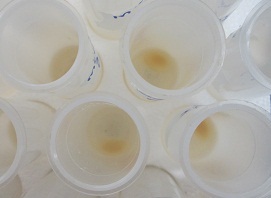

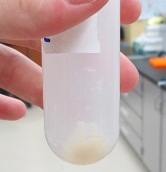


**Supplementary Figure 3**

Supplementary Fig: 3. Photo of the slightly yellow- translucent EPS extracted and purified from soils in a 25 mL tube.

Supplementary Table 1a,b. The mean for each microbial AA g^-1^ soil in both Marietta and Sumter soils at different levels of matric potential and amendment. Overall, Marietta had greater concentrations of AA. Generally, drying decreased AA and amendment increased AA content. AA with the most significant drying effects are shown in graphical form (Figures 2-4; Supp Fig 1-2.). Gln, GABA, and Arg were greater in Sumter, while the remaining AA were similar or significantly greater in Marietta. On a mol% basis, however, Asp, Thr Ala and Val, were significantly greater in Mariettta relative to Sumter.

| ng/g soil | No amendment | | | C only amendment | | | C and N amendment | | |
| --- | --- | --- | --- | --- | --- | --- | --- | --- | --- |
|  | Moist | Intermediately  dry | Dry | Moist | Intermediately  dry | Dry | Moist | Intermediately  dry | Dry |
| ASP | 1075.08 | 1325.57 | 884.45 | 1795.50 | 1589.35 | 899.97 | 1642.55 | 1369.90 | 1075.08 |
| Glu | 7136.85 | 8829.80 | 6583.15 | 9645.65 | 9145.85 | 6401.85 | 10221.40 | 9420.25 | 7915.95 |
| Ser+Asn | 383.47 | 470.45 | 314.29 | 606.84 | 549.51 | 304.41 | 589.05 | 482.31 | 349.87 |
| Gly | 709.70 | 849.88 | 589.54 | 1201.60 | 1086.45 | 608.31 | 1132.76 | 916.22 | 715.95 |
| Gln | 172.77 | 163.03 | 199.53 | 77.87 | 104.63 | 160.60 | 138.70 | 167.90 | 206.83 |
| His | 28.71 | 36.99 | 13.97 | 63.11 | 50.18 | 17.54 | 58.46 | 44.75 | 26.90 |
| Arg | 180.01 | 235.17 | 150.97 | 270.01 | 238.07 | 139.36 | 284.53 | 240.98 | 180.01 |
| Cit+Tau | 170.23 | 187.75 | 177.74 | 187.75 | 185.25 | 162.72 | 207.78 | 172.73 | 212.78 |
| Thr | 817.13 | 950.02 | 684.25 | 1217.77 | 1140.42 | 688.22 | 1205.87 | 959.93 | 763.58 |
| Ala | 2190.38 | 2536.38 | 1860.71 | 3264.03 | 2974.46 | 1833.98 | 3130.38 | 2661.12 | 2141.37 |
| GABA | 166.68 | 175.27 | 92.79 | 183.86 | 154.65 | 85.23 | 194.17 | 216.51 | 109.97 |
| Pro | 375.99 | 496.85 | 306.93 | 661.83 | 571.66 | 347.22 | 573.58 | 573.58 | 408.61 |
| Tyr | 305.02 | 377.50 | 350.32 | 437.90 | 419.78 | 389.58 | 474.14 | 410.72 | 477.16 |
| Val | 1774.07 | 2055.11 | 1719.42 | 2650.36 | 2414.21 | 1682.34 | 2533.26 | 2086.33 | 1957.52 |
| Met | 218.83 | 288.45 | 196.45 | 375.49 | 315.81 | 203.91 | 365.54 | 278.51 | 243.69 |
| Orn | 89.36 | 109.59 | 76.15 | 148.93 | 126.45 | 75.03 | 151.74 | 106.78 | 94.98 |
| Ile | 1045.23 | 1209.23 | 1005.87 | 1613.76 | 1469.44 | 1025.55 | 1552.53 | 1226.72 | 1158.93 |
| Lys | 85.28 | 119.40 | 42.64 | 168.13 | 146.20 | 42.32 | 163.26 | 131.58 | 66.03 |
| Leu | 1891.47 | 2151.68 | 1766.83 | 2757.39 | 2484.05 | 1727.47 | 2621.81 | 2114.51 | 1976.75 |
| Phe | 754.41 | 875.56 | 710.36 | 1117.85 | 988.45 | 715.87 | 1076.55 | 859.04 | 875.56 |
| Trp | 129.33 | 153.15 | 139.54 | 176.97 | 142.94 | 0.00 | 156.55 | 129.33 | 132.73 |

Supplementary Table 1b: The concentration of individual microbial AA g^-1^ soil in Sumter soils at different levels of matric potential and nutrient amendment. The values indicate the mean of 3 replicates of each treatment.

| ng/g soil | No amendment | | | C amendment | | | C and N amendment | | |
| --- | --- | --- | --- | --- | --- | --- | --- | --- | --- |
|  | Moist | Intermediately  dry | Dry | Moist | Intermediately  dry | Dry | Moist | Intermediately  dry | Dry |
| ASP | 220.34 | 168.91 | 91.33 | 188.86 | 172.01 | 132.56 | 187.97 | 252.70 | 310.78 |
| Glu | 3528.00 | 3204.60 | 1901.20 | 3663.73 | 3748.99 | 2914.52 | 4346.79 | 5207.23 | 3477.04 |
| Ser+Asn | 192.92 | 166.44 | 110.30 | 178.30 | 172.37 | 156.95 | 212.69 | 278.31 | 309.94 |
| Gly | 167.22 | 125.67 | 75.35 | 129.92 | 120.41 | 118.41 | 135.43 | 194.76 | 141.44 |
| Gln | 533.87 | 697.88 | 702.26 | 550.91 | 658.46 | 884.27 | 987.93 | 864.32 | 1234.19 |
| His | 2.83 | 2.53 | 0.93 | 2.33 | 0.88 | 0.88 | 2.02 | 3.83 | 3.98 |
| Arg | 415.76 | 414.60 | 309.50 | 348.98 | 361.76 | 338.53 | 407.63 | 389.05 | 389.63 |
| Cit+Tau | 124.67 | 101.64 | 105.14 | 42.26 | 46.56 | 91.62 | 144.19 | 181.24 | 214.29 |
| Thr | 270.13 | 217.37 | 147.96 | 230.86 | 223.32 | 209.84 | 264.58 | 396.27 | 317.73 |
| Ala | 748.14 | 554.80 | 373.03 | 602.02 | 588.95 | 498.07 | 675.38 | 887.44 | 596.97 |
| GABA | 435.77 | 335.42 | 170.46 | 589.39 | 597.29 | 311.02 | 1459.21 | 1463.68 | 787.34 |
| Pro | 58.32 | 42.20 | 32.23 | 52.18 | 51.41 | 47.19 | 59.85 | 77.88 | 115.10 |
| Tyr | 246.43 | 220.46 | 175.76 | 134.69 | 131.07 | 219.86 | 212.61 | 308.04 | 340.66 |
| Val | 427.42 | 293.53 | 277.53 | 325.54 | 326.32 | 398.92 | 359.50 | 509.78 | 487.53 |
| Met | 214.35 | 175.06 | 129.80 | 134.78 | 129.31 | 148.70 | 213.85 | 282.49 | 217.83 |
| Orn | 44.40 | 30.35 | 13.09 | 24.17 | 24.73 | 19.28 | 33.72 | 50.58 | 32.03 |
| Ile | 240.97 | 163.56 | 156.13 | 177.99 | 181.49 | 237.03 | 201.61 | 294.33 | 291.70 |
| Lys | 73.10 | 62.87 | 30.21 | 69.69 | 59.45 | 49.22 | 75.54 | 92.59 | 63.35 |
| Leu | 710.67 | 523.49 | 427.71 | 485.00 | 470.13 | 583.40 | 634.13 | 875.98 | 734.28 |
| Phe | 325.99 | 236.79 | 199.34 | 100.22 | 101.32 | 188.88 | 275.88 | 393.73 | 391.52 |
| Trp | 0.00 | 0.00 | 0.00 | 0.00 | 21.76 | 54.45 | 0.00 | 0.00 | 0.00 |

Supplementary Table 2a,b. The mean for each AA g^-1^ soil MBC in both Marietta (a.) and Sumter (b.) soils at different levels of matric potential and amendment. Generally, drying decreased AA and amendment increased AA content. There were not significant increases in microbial AA due to drying based on MBC basis, however, AA almost always declined significantly or did not change due to drying. Amendment usually significantly increased AA content.

| \| ng g^-1^ MBC \| No amendment    Moist Intermediate dry Dry \| \| \| \| \| \| C amendment  Moist Intermediate dry Dry \| \| \| \| \| \| C and N amendment  Moist Intermediate dry Dry \| \| \| \| \| \| \| --- \| --- \| --- \| --- \| --- \| --- \| --- \| --- \| --- \| --- \| --- \| --- \| --- \| --- \| --- \| --- \| --- \| --- \| --- \| \|  \| AVG \| SE \| AVG \| SE \| AVG \| SE \| AVG \| SE \| AVG \| SE \| AVG \| SE \| AVG \| SE \| AVG \| SE \| AVG \| SE \| \| ASP \| 2.47 \| 0.01 \| 2.62 \| 0.11 \| 1.53 \| 0.13 \| 3.79 \| 0.14 \| 3.43 \| 0.53 \| 1.85 \| 0.15 \| 3.00 \| 0.30 \| 2.32 \| 0.07 \| 1.45 \| 0.14 \| \| Glu \| 16.50 \| 0.52 \| 17.45 \| 0.50 \| 11.38 \| 0.86 \| 20.32 \| 0.98 \| 19.39 \| 1.70 \| 13.21 \| 1.23 \| 18.79 \| 1.76 \| 15.97 \| 0.38 \| 10.64 \| 1.00 \| \| Asn \| 0.89 \| 0.03 \| 0.93 \| 0.04 \| 0.54 \| 0.05 \| 1.28 \| 0.07 \| 1.18 \| 0.14 \| 0.62 \| 0.06 \| 1.08 \| 0.13 \| 0.82 \| 0.02 \| 0.47 \| 0.02 \| \| Gly \| 1.64 \| 0.03 \| 1.68 \| 0.06 \| 1.02 \| 0.08 \| 2.53 \| 0.12 \| 2.33 \| 0.30 \| 1.25 \| 0.13 \| 2.07 \| 0.27 \| 1.55 \| 0.02 \| 0.96 \| 0.08 \| \| Gln \| 0.40 \| 0.03 \| 0.33 \| 0.04 \| 0.35 \| 0.04 \| 0.16 \| 0.01 \| 0.22 \| 0.01 \| 0.33 \| 0.03 \| 0.26 \| 0.06 \| 0.29 \| 0.01 \| 0.28 \| 0.02 \| \| His \| 0.07 \| 0.00 \| 0.07 \| 0.01 \| 0.02 \| 0.01 \| 0.13 \| 0.01 \| 0.11 \| 0.02 \| 0.03 \| 0.01 \| 0.11 \| 0.02 \| 0.08 \| 0.00 \| 0.04 \| 0.00 \| \| Arg \| 0.42 \| 0.03 \| 0.47 \| 0.04 \| 0.26 \| 0.01 \| 0.57 \| 0.04 \| 0.51 \| 0.06 \| 0.30 \| 0.01 \| 0.52 \| 0.06 \| 0.41 \| 0.01 \| 0.24 \| 0.02 \| \| Tau \| 0.39 \| 0.01 \| 0.37 \| 0.01 \| 0.31 \| 0.02 \| 0.40 \| 0.00 \| 0.40 \| 0.06 \| 0.34 \| 0.03 \| 0.38 \| 0.02 \| 0.29 \| 0.02 \| 0.28 \| 0.01 \| \| Thr \| 1.89 \| 0.06 \| 1.88 \| 0.08 \| 1.18 \| 0.08 \| 2.57 \| 0.14 \| 2.43 \| 0.26 \| 1.42 \| 0.13 \| 2.21 \| 0.25 \| 1.63 \| 0.04 \| 1.02 \| 0.06 \| \| Ala \| 5.05 \| 0.12 \| 5.02 \| 0.18 \| 3.22 \| 0.18 \| 6.88 \| 0.34 \| 6.35 \| 0.73 \| 3.80 \| 0.30 \| 5.72 \| 0.65 \| 4.51 \| 0.08 \| 2.88 \| 0.26 \| \| GABA \| 0.38 \| 0.03 \| 0.35 \| 0.03 \| 0.16 \| 0.00 \| 0.39 \| 0.06 \| 0.32 \| 0.02 \| 0.17 \| 0.03 \| 0.36 \| 0.04 \| 0.37 \| 0.00 \| 0.15 \| 0.03 \| \| Pro \| 0.85 \| 0.08 \| 0.98 \| 0.08 \| 0.53 \| 0.06 \| 1.39 \| 0.07 \| 1.24 \| 0.18 \| 0.71 \| 0.09 \| 1.04 \| 0.22 \| 0.97 \| 0.03 \| 0.55 \| 0.07 \| \| Tyr \| 0.71 \| 0.05 \| 0.75 \| 0.04 \| 0.61 \| 0.03 \| 0.92 \| 0.05 \| 0.89 \| 0.08 \| 0.81 \| 0.06 \| 0.87 \| 0.09 \| 0.70 \| 0.01 \| 0.64 \| 0.04 \| \| Val \| 4.10 \| 0.10 \| 4.07 \| 0.16 \| 2.98 \| 0.15 \| 5.58 \| 0.28 \| 5.16 \| 0.62 \| 3.49 \| 0.25 \| 4.63 \| 0.56 \| 3.54 \| 0.06 \| 2.63 \| 0.21 \| \| Met \| 0.51 \| 0.03 \| 0.58 \| 0.05 \| 0.34 \| 0.03 \| 0.79 \| 0.09 \| 0.66 \| 0.07 \| 0.41 \| 0.07 \| 0.66 \| 0.13 \| 0.47 \| 0.04 \| 0.33 \| 0.07 \| \| Orn \| 0.21 \| 0.00 \| 0.22 \| 0.01 \| 0.13 \| 0.01 \| 0.31 \| 0.02 \| 0.27 \| 0.04 \| 0.15 \| 0.02 \| 0.28 \| 0.05 \| 0.18 \| 0.00 \| 0.13 \| 0.02 \| \| Ile \| 2.41 \| 0.07 \| 2.39 \| 0.09 \| 1.74 \| 0.09 \| 3.40 \| 0.17 \| 3.14 \| 0.38 \| 2.12 \| 0.15 \| 2.84 \| 0.35 \| 2.08 \| 0.04 \| 1.56 \| 0.12 \| \| Lys \| 0.20 \| 0.01 \| 0.24 \| 0.00 \| 0.07 \| 0.01 \| 0.35 \| 0.03 \| 0.31 \| 0.04 \| 0.08 \| 0.03 \| 0.30 \| 0.04 \| 0.22 \| 0.01 \| 0.09 \| 0.01 \| \| Leu \| 4.38 \| 0.16 \| 4.26 \| 0.18 \| 3.07 \| 0.13 \| 5.81 \| 0.28 \| 5.31 \| 0.61 \| 3.58 \| 0.24 \| 4.79 \| 0.57 \| 3.58 \| 0.04 \| 2.65 \| 0.21 \| \| Phe \| 1.75 \| 0.08 \| 1.74 \| 0.08 \| 1.23 \| 0.06 \| 2.36 \| 0.12 \| 2.11 \| 0.24 \| 1.47 \| 0.13 \| 1.97 \| 0.25 \| 1.46 \| 0.02 \| 1.17 \| 0.10 \| \| Trp \| 0.30 \| 0.01 \| 0.31 \| 0.04 \| 0.24 \| 0.01 \| 0.37 \| 0.03 \| 0.31 \| 0.05 \| 0.00 \| 0.00 \| 0.29 \| 0.02 \| 0.22 \| 0.03 \| 0.18 \| 0.01 \| |
| --- | --- | --- | --- | --- | --- | --- | --- | --- | --- | --- | --- | --- | --- | --- | --- | --- | --- | --- | --- | --- | --- | --- | --- | --- | --- | --- | --- | --- | --- | --- | --- | --- | --- | --- | --- | --- | --- | --- | --- | --- | --- | --- | --- | --- | --- | --- | --- | --- | --- | --- | --- | --- | --- | --- | --- | --- | --- | --- | --- | --- | --- | --- | --- | --- | --- | --- | --- | --- | --- | --- | --- | --- | --- | --- | --- | --- | --- | --- | --- | --- | --- | --- | --- | --- | --- | --- | --- | --- | --- | --- | --- | --- | --- | --- | --- | --- | --- | --- | --- | --- | --- | --- | --- | --- | --- | --- | --- | --- | --- | --- | --- | --- | --- | --- | --- | --- | --- | --- | --- | --- | --- | --- | --- | --- | --- | --- | --- | --- | --- | --- | --- | --- | --- | --- | --- | --- | --- | --- | --- | --- | --- | --- | --- | --- | --- | --- | --- | --- | --- | --- | --- | --- | --- | --- | --- | --- | --- | --- | --- | --- | --- | --- | --- | --- | --- | --- | --- | --- | --- | --- | --- | --- | --- | --- | --- | --- | --- | --- | --- | --- | --- | --- | --- | --- | --- | --- | --- | --- | --- | --- | --- | --- | --- | --- | --- | --- | --- | --- | --- | --- | --- | --- | --- | --- | --- | --- | --- | --- | --- | --- | --- | --- | --- | --- | --- | --- | --- | --- | --- | --- | --- | --- | --- | --- | --- | --- | --- | --- | --- | --- | --- | --- | --- | --- | --- | --- | --- | --- | --- | --- | --- | --- | --- | --- | --- | --- | --- | --- | --- | --- | --- | --- | --- | --- | --- | --- | --- | --- | --- | --- | --- | --- | --- | --- | --- | --- | --- | --- | --- | --- | --- | --- | --- | --- | --- | --- | --- | --- | --- | --- | --- | --- | --- | --- | --- | --- | --- | --- | --- | --- | --- | --- | --- | --- | --- | --- | --- | --- | --- | --- | --- | --- | --- | --- | --- | --- | --- | --- | --- | --- | --- | --- | --- | --- | --- | --- | --- | --- | --- | --- | --- | --- | --- | --- | --- | --- | --- | --- | --- | --- | --- | --- | --- | --- | --- | --- | --- | --- | --- | --- | --- | --- | --- | --- | --- | --- | --- | --- | --- | --- | --- | --- | --- | --- | --- | --- | --- | --- | --- | --- | --- | --- | --- | --- | --- | --- | --- | --- | --- | --- | --- | --- | --- | --- | --- | --- | --- | --- | --- | --- | --- | --- | --- | --- | --- | --- | --- | --- | --- | --- | --- | --- | --- | --- | --- | --- | --- | --- | --- | --- | --- | --- | --- | --- | --- | --- | --- | --- | --- | --- | --- | --- | --- | --- | --- | --- | --- | --- | --- | --- | --- | --- | --- | --- | --- | --- | --- | --- | --- | --- | --- | --- | --- | --- | --- | --- | --- |

Supplementary Table 2b: The mean for each AA g^-1^ soil MBC in Sumter soil at different levels of matric potential and nutrient amendment.

| ng g^-1^ MBC | No amendment    Moist Intermediate dry Dry | | | | | | C amendment  Moist Intermediate dry Dry | | | | | | C and N amendment  Moist Intermediate dry Dry | | | | | |
| --- | --- | --- | --- | --- | --- | --- | --- | --- | --- | --- | --- | --- | --- | --- | --- | --- | --- | --- |
|  | AVG | SE | AVG | SE | AVG | SE | AVG | SE | AVG | SE | AVG | SE | AVG | SE | AVG | SE | AVG | SE |
| ASP | 0.76 | 0.07 | 0.64 | 0.09 | 0.25 | 0.04 | 0.64 | 0.06 | 0.45 | 0.03 | 0.26 | 0.01 | 0.54 | 0.07 | 0.57 | 0.05 | 0.50 | 0.24 |
| Glu | 12.20 | 1.08 | 12.18 | 1.56 | 5.21 | 0.82 | 12.36 | 0.51 | 9.88 | 0.21 | 5.69 | 0.39 | 12.48 | 0.97 | 11.76 | 1.01 | 5.82 | 0.22 |
| Ser+Asn | 0.67 | 0.10 | 0.63 | 0.07 | 0.30 | 0.05 | 0.60 | 0.04 | 0.46 | 0.01 | 0.31 | 0.02 | 0.61 | 0.06 | 0.63 | 0.04 | 0.50 | 0.17 |
| Gly | 0.58 | 0.04 | 0.47 | 0.05 | 0.21 | 0.04 | 0.44 | 0.04 | 0.32 | 0.00 | 0.23 | 0.01 | 0.39 | 0.04 | 0.44 | 0.03 | 0.24 | 0.00 |
| Gln | 1.86 | 0.39 | 2.73 | 0.68 | 1.90 | 0.21 | 1.83 | 0.19 | 1.74 | 0.10 | 1.75 | 0.25 | 2.85 | 0.05 | 1.95 | 0.27 | 2.08 | 0.14 |
| His | 0.01 | 0.00 | 0.01 | 0.00 | 0.00 | 0.00 | 0.01 | 0.00 | 0.00 | 0.00 | 0.00 | 0.00 | 0.01 | 0.00 | 0.01 | 0.00 | 0.01 | 0.00 |
| Arg | 1.44 | 0.19 | 1.62 | 0.39 | 0.85 | 0.13 | 1.17 | 0.03 | 0.96 | 0.11 | 0.66 | 0.04 | 1.17 | 0.07 | 0.88 | 0.08 | 0.66 | 0.03 |
| Cit+Tau | 0.43 | 0.02 | 0.38 | 0.04 | 0.29 | 0.05 | 0.14 | 0.03 | 0.12 | 0.00 | 0.18 | 0.01 | 0.41 | 0.03 | 0.41 | 0.02 | 0.36 | 0.02 |
| Thr | 0.93 | 0.15 | 0.81 | 0.09 | 0.41 | 0.08 | 0.78 | 0.05 | 0.59 | 0.02 | 0.41 | 0.03 | 0.76 | 0.11 | 0.89 | 0.05 | 0.53 | 0.04 |
| Ala | 2.58 | 0.21 | 2.06 | 0.19 | 1.03 | 0.18 | 2.04 | 0.16 | 1.55 | 0.02 | 0.97 | 0.05 | 1.94 | 0.17 | 2.00 | 0.13 | 1.00 | 0.05 |
| GABA | 1.51 | 0.12 | 1.26 | 0.12 | 0.46 | 0.05 | 2.01 | 0.32 | 1.58 | 0.12 | 0.61 | 0.04 | 4.20 | 0.40 | 3.32 | 0.46 | 1.31 | 0.14 |
| Pro | 0.20 | 0.01 | 0.16 | 0.01 | 0.09 | 0.02 | 0.18 | 0.01 | 0.14 | 0.00 | 0.09 | 0.00 | 0.17 | 0.01 | 0.18 | 0.03 | 0.18 | 0.08 |
| Tyr | 0.85 | 0.05 | 0.82 | 0.10 | 0.48 | 0.09 | 0.45 | 0.02 | 0.35 | 0.04 | 0.43 | 0.02 | 0.61 | 0.07 | 0.69 | 0.03 | 0.57 | 0.02 |
| Val | 1.48 | 0.06 | 1.08 | 0.11 | 0.77 | 0.14 | 1.10 | 0.09 | 0.86 | 0.02 | 0.78 | 0.02 | 1.03 | 0.08 | 1.15 | 0.05 | 0.81 | 0.03 |
| Met | 0.74 | 0.09 | 0.65 | 0.04 | 0.36 | 0.06 | 0.46 | 0.04 | 0.34 | 0.01 | 0.29 | 0.02 | 0.61 | 0.06 | 0.64 | 0.03 | 0.36 | 0.02 |
| Orn | 0.15 | 0.02 | 0.11 | 0.01 | 0.04 | 0.01 | 0.08 | 0.01 | 0.07 | 0.00 | 0.04 | 0.00 | 0.10 | 0.01 | 0.11 | 0.00 | 0.05 | 0.01 |
| Ile | 0.83 | 0.05 | 0.60 | 0.07 | 0.43 | 0.09 | 0.60 | 0.05 | 0.48 | 0.01 | 0.46 | 0.02 | 0.58 | 0.05 | 0.66 | 0.03 | 0.49 | 0.01 |
| Lys | 0.25 | 0.01 | 0.25 | 0.06 | 0.08 | 0.02 | 0.23 | 0.01 | 0.16 | 0.01 | 0.10 | 0.01 | 0.22 | 0.03 | 0.21 | 0.02 | 0.11 | 0.00 |
| Leu | 2.45 | 0.12 | 1.94 | 0.16 | 1.18 | 0.21 | 1.64 | 0.14 | 1.24 | 0.02 | 1.14 | 0.05 | 1.82 | 0.14 | 1.98 | 0.09 | 1.23 | 0.05 |
| Phe | 1.13 | 0.05 | 0.88 | 0.08 | 0.55 | 0.10 | 0.34 | 0.05 | 0.27 | 0.01 | 0.37 | 0.01 | 0.79 | 0.07 | 0.89 | 0.04 | 0.65 | 0.05 |
| Trp | 0.00 | 0.00 | 0.00 | 0.00 | 0.00 | 0.00 | 0.00 | 0.00 | 0.06 | 0.03 | 0.11 | 0.01 | 0.00 | 0.00 | 0.00 | 0.00 | 0.00 | 0.00 |
